# Crosstalk between the IGF-1R/AKT/mTORC1 pathway and the tumor suppressors p53 and p27 determines cisplatin sensitivity and limits the effectiveness of an IGF-1R pathway inhibitor

**DOI:** 10.18632/oncotarget.8484

**Published:** 2016-03-30

**Authors:** Batzaya Davaadelger, Lei Duan, Ricardo E. Perez, Steven Gitelis, Carl G. Maki

**Affiliations:** ^1^ Department of Anatomy and Cell Biology, Rush University Medical Center, Chicago, IL, USA; ^2^ Section of Orthopedic Oncology, Department of Orthopedic Surgery, Rush University, Medical Center, Chicago, IL, USA

**Keywords:** p53, p27, IGF-1R/AKT/mTORC1, cisplatin

## Abstract

The insulin-like growth factor-1 receptor (IGF-1R) signaling pathway is aberrantly activated in multiple cancers and can promote proliferation and chemotherapy resistance. Multiple IGF-1R inhibitors have been developed as potential therapeutics. However, these inhibitors have failed to increase patient survival when given alone or in combination with chemotherapy agents. The reason(s) for the disappointing clinical effect of these inhibitors is not fully understood. Cisplatin (CP) activated the IGF-1R/AKT/mTORC1 pathway and stabilized p53 in osteosarcoma (OS) cells. p53 knockdown reduced IGF-1R/AKT/mTORC1 activation by CP, and IGF-1R inhibition reduced the accumulation of p53. These data demonstrate positive crosstalk between p53 and the IGF-1R/AKT/mTORC1 pathway in response to CP. Further studies showed the effect of IGF-1R inhibition on CP response is dependent on p53 status. In p53 wild-type cells treated with CP, IGF-1R inhibition increased p53s apoptotic function but reduced p53-dependent senescence, and had no effect on long term survival. In contrast, in p53-null/knockdown cells, IGF-1R inhibition reduced apoptosis in response to CP and increased long term survival. These effects were due to p27 since IGF-1R inhibition stabilized p27 in CP-treated cells, and p27 depletion restored apoptosis and reduced long term survival. Together, the results demonstrate 1) p53 expression determines the effect of IGF-1R inhibition on cancer cell CP response, and 2) crosstalk between the IGF-1R/AKT/mTORC1 pathway and p53 and p27 can reduce cancer cell responsiveness to chemotherapy and may ultimately limit the effectiveness of IGF-1R pathway inhibitors in the clinic.

## INTRODUCTION

The IGF-1R/IR/AKT/mTORC1 pathway is aberrantly activated in multiple cancer types and can promote and/or regulate proliferation and chemotherapy resistance [1–6]. Ligands IGF1 and IGF2 bind the receptor IGF-1R stimulating its auto-phosphorylation on tyrosines. This leads to recruitment/activation of adaptor proteins (e.g. IRS1). The kinase AKT is subsequently recruited and activated by phosphorylation at two sites: Serine 473 (S473) is phosphorylated by mTORC2 and Threonine 308 (T308) is phosphorylated by PDK1. Activated AKT can promote survival by inhibiting and/or promoting the activity of various pro/anti apoptotic factors [1, 4–6]. In addition, AKT can also promote the degradation of p27 [7, 8], a cyclin dependent kinase inhibitor that can arrest cells in G1-phase by binding and inhibiting G1-phase cyclin-cdk complexes [9]. The mTORC1 kinase complex is activated downstream of AKT and promotes survival, metabolism, growth, and protein synthesis/translation by phosphorylation of substrates (e.g. S6K) [10, 11]. Notably, activated S6K (pS6K) can also inhibit signaling from IGF-1R to AKT by promoting the degradation of IRS [12, 13]. Multiple inhibitors of the IGF-1R/IR/AKT/mTORC1 pathway have been developed and are in various phases of clinical testing [14–17]. However, while these inhibitors have shown promise in pre-clinical studies they have largely failed to increase long-term patient survival [14, 16, 17]. The reason(s) for the disappointing clinical effect of IGF-1R/IR/AKT/mTORC1 inhibitors is not fully understood.

The tumor suppressor protein p53 is a transcription factor and key regulator of the cellular response to DNA damage and chemotherapy. Wild-type p53 is normally expressed at low levels and inactive due to MDM2, an E3 ubiquitin-ligase that binds p53 and promotes its degradation [18, 19]. However, the p53 protein is stabilized and becomes activated in response to DNA damage that results, for example, from chemotherapeutic drug treatment or radiation. Stabilization of p53 is believed to result from DNA damage-induced post-translational modifications that disrupt p53-MDM2 binding. The effect of stabilizing and activating p53 can vary and may depend on cell-type, the level of DNA damage, and the ability of cells to undergo DNA repair [20–23]. For example, in response to transient or low levels of DNA damage p53 can trigger reversible arrests in the G1 and G2-phases of the cell cycle [24]. The G1 arrest is mediated by p21, a p53-responsive gene product that, like p27, can arrest cells in G1 by binding to and inhibiting the activity of G1-phase cyclin-cdk complexes [9, 25–27]. p53 is not required to initiate the G2 arrest after DNA damage but functions to maintain the arrest. G2-arrest maintenance by p53 may result from downregulation of *Cyclin B1*, *CDC2,* and other genes, or by increased expression of 14-3-3σ, which can sequester and inhibit Cyclin B-CDC2 complexes [28, 29]. Notably, the reversible G1 and G2 arrests mediated by p53 could increase cancer cell survival in response to radiation or chemotherapeutic drug treatment by allowing cells time to repair their DNA before proceeding with either replicative DNA synthesis or mitosis. In contrast, when DNA damage is prolonged or excessive, activated p53 can trigger either a permanent, senescent arrest that is also dependent on p21 [30–32] or apoptotic death by inducing expression of pro-apoptotic factors like Puma and Noxa [23, 33, 34]. The molecular factors and/or pathways that control the choice of response to p53 (e.g. survival, senescence, or apoptosis) are largely unknown.

There is abundant cross-talk between the p53 and IGF-1R/AKT/mTORC1 pathways which could influence the cellular response to DNA damage and chemotherapy [35–39]. Most studies suggest p53 can inhibit IGF-1R/AKT/mTORC1 signaling and, conversely, that IGF-1R/AKT/mTORC1 activation can inhibit p53 [36–38, 40–42]. Evidence p53 can inhibit the IGF-1R/AKT/mTORC1 pathway includes reports that p53 can repress expression of the *IGF-1R* and *IGF1* genes [43–45] and induce expression of IGF-BP3, a factor that can sequester and inhibit IGF1 [46, 47]. Evidence IGF-1R/AKT activation can inhibit p53 includes studies from Mayo and colleagues in which it was found AKT activated downstream of IGF1 promoted the ability of MDM2 to degrade p53 [48]. However, there are also studies that support positive crosstalk between p53 and the IGF-1R/AKT/mTORC1 pathway. For example, p53 can inhibit mTORC1 and this inhibition may increase AKT activation by releasing feedback inhibition of the pathway that is normally mediated by pS6K [13, 49]. Furthermore, Blattner and colleagues reported that AKT activated by ionizing radiation (IR) promoted the stabilization of p53 [50]. Finally, there are also reports that activated mTORC1 can promote p53 protein synthesis [51, 52]. In summary, there is evidence for both positive and negative crosstalk between p53 and IGF-1R/AKT/mTORC1 signaling. The impact of this crosstalk on DNA damage responses and cell fate decisions downstream of p53 is unknown.

In the current report we examined crosstalk between p53 and IGF-1R/AKT/mTORC1 pathway in response to the common chemotherapeutic agent cisplatin (CP), and how this crosstalk influences cell fate. CP treatment activated the IGF-1R/AKT/mTORC1 pathway and induced p53 in multiple OS cell lines and primary OS cells. IGF-1R/AKT/mTORC1 inhibitors reduced p53 accumulation in CP-treated cells, and p53 knockdown reduced IGF-1R/AKT/mTORC1 activation. These results indicate positive crosstalk between p53 and the IGF-1R/AKT/mTORC1 signaling pathway in response to CP. In p53 wild-type (WT) OS cells, IGF-1R inhibition increased p53-dependent apoptosis but reduced p53-dependent senescence, and therefore had no effect on long-term survival (colony formation). In contrast, IGF-1R inhibition promoted long term survival of OS cells that lack p53 or in which p53 was knocked down. This effect was due at least in part to p27 since IGF-1R inhibition stabilized p27 in CP-treated cells, and p27 depletion restored apoptosis sensitivity and reduced long-term survival. The results demonstrate that IGF-1R inhibition has different effects on cancer cell response to CP depending on whether the cells express or do not express p53. Further, the results demonstrate crosstalk between the IGF-1R/AKT/mTORC1 pathway and the tumor suppressors p53 and p27 that regulate cell fate decisions in response to p53 and that can determine cancer cell responsiveness to chemotherapy. These findings have potential implications regarding the use of IGF-1R/IR inhibitors against p53 wild-type or p53 mutant/null cancer cells.

## RESULTS

### Cisplatin activates the IGF-1R/AKT pathway in osteosarcoma cells, and this activation contributes to the accumulation of p53

**Figure 1:**
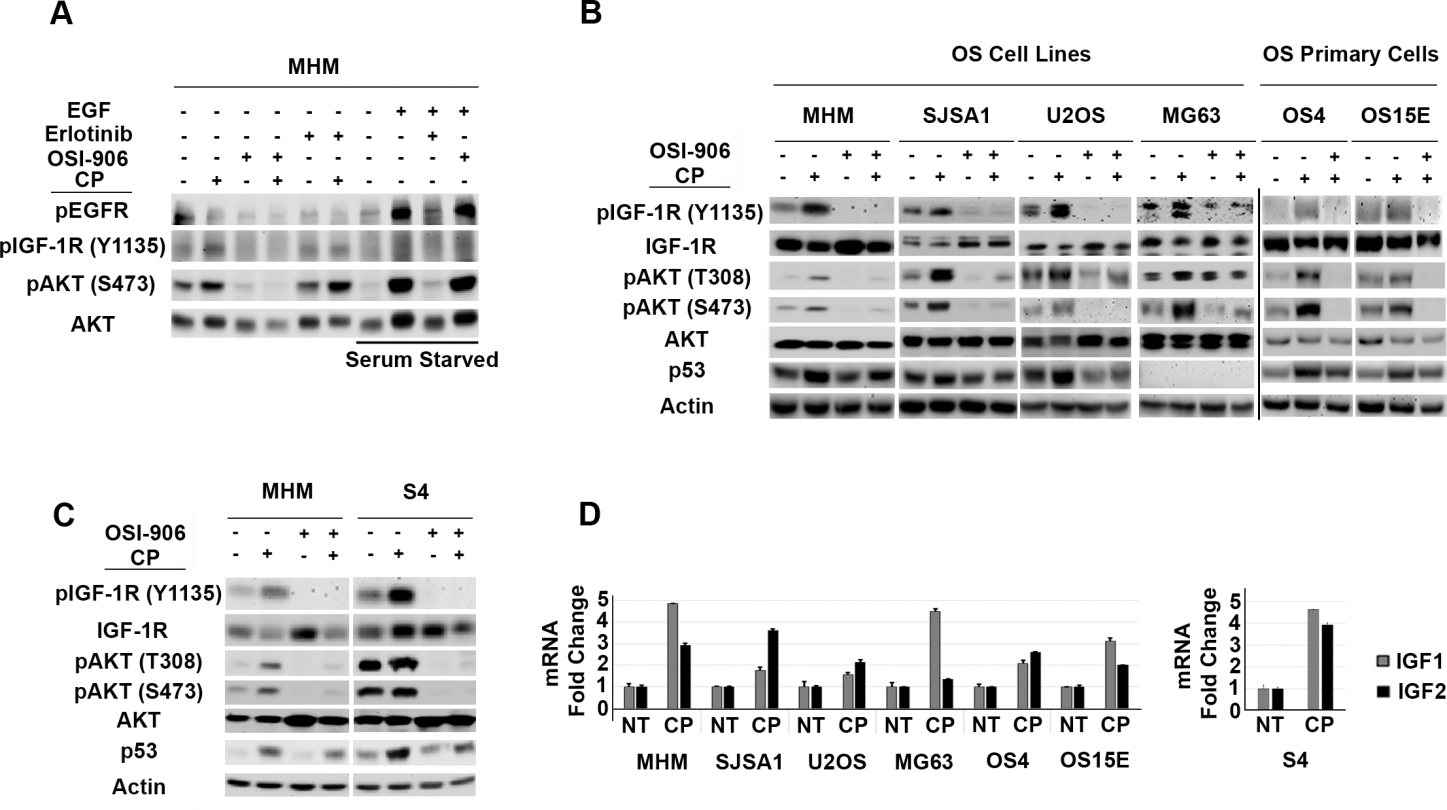
Cisplatin induces IGF-1R/AKT activation in osteosarcoma cells and it is accompanied by increased IGF 1/2 gene expression (**A**) MHM cells were treated with CP (10 μM) alone or in combination with OSI-906 (5 μM) or Erlotinib (10 μM) for 48 hours and lysates were immunoblotted with indicated antibodies. EGFR immuno-precipitates were probed with anti-phospho tyrosine antibody. In lanes marked “Serum Starved”, MHM cells were serum starved for 24 hours and stimulated with EGF (10 ng/ml) for 10 minutes. Lysates were collected and immunoblotted with indicated antibodies. (**B**) OS cell lines and primary OS cells were treated with CP (10 μM) alone, OSI-906 (5 μM) alone or with CP in combination with OSI-906 for 48 hours and whole cell lysates were immunoblotted with the indicated antibodies. (**C**) MHM and S4 (CP resistant MHM derivatives) cells were treated with CP (10 μM) alone or in combination with OSI-906 (5 μM) for 48 hours and lysates were immunoblotted with indicated antibodies. (**D**) IGF1 and IGF2 mRNA levels in untreated cells and cells treated with 10 μM CP for 48 hours were determined by qRT-PCR. The level of each mRNA transcript in untreated cells was considered “1.0” and all the other values were plotted relative to it. Shown are the mean results of three experiments, *bars*, Standard error (SE).

In our previous studies we found that AKT was activated in cisplatin (CP)-treated osteosarcoma (OS) cells, and that AKT inhibitors could sensitize p53 wild-type OS cells to CP [53]. We wished to test if AKT activation in response to CP was IGF-1R/IR-dependent. To this end, the OS cell line MHM was treated for 48 hours with CP alone or CP plus either OSI-906 (IGF-1R/IR inhibitor) or Erlotinib (EGFR inhibitor). Levels of pIGF-1R and pAKT were then determined by immunoblot, and pEGFR levels determined by probing EGFR immuno-precipitates with anti-phospho tyrosine antibody. As shown in Figure 1A, pIGF-1R (Y1135) and pAKT (S473) levels were increased in CP-treated MHM cells, and these increases were blocked by OSI-906 but not blocked by Erlotinib. In serum starved MHM cells, exogenous EGF increased levels of pEGFR and pAKT(S473) and this effect was blocked by Erlotinib but not by OSI-906. The results indicate that CP induces pAKT(S473) in MHM cells via IGF-1R/IR and not EGFR activation.

Next, we wished to ask two questions: 1) Does CP activate the IGF-1R/AKT pathway in multiple OS cells and 2) Does IGF-1R/AKT pathway affect p53 levels in response to CP? To address these questions, we tested the effect of CP on the IGF-1R/AKT pathway and p53 in multiple OS cell lines and primary OS cells derived from patient tumor resections. In each of the OS cell lines and primary OS cultures we found that CP treatment increased phosphorylation (activation) of IGF-1R and AKT, and that this effect was blocked by OSI-906 (Figure 1B). These results indicate CP activation of IGF-1R/AKT signaling is seen in multiple OS cells and is not specific to MHM cells. p53 was induced by CP in all the cell lines except MG63, which are p53-null. Importantly, co-treatment with OSI-906 reduced p53 accumulation in CP-treated OS cells, suggesting that IGF-1R/AKT pathway activation contributes to p53 accumulation (Figure 1B). We isolated MHM cells that survived repeated CP exposure. These cells (referred to as S4 cells) are resistant to CP induced apoptosis compared to MHM (Supplementary Figure 1) and have heightened basal and CP induced levels of pIGF-1R (Y1135) and pAKT (S473 and T308) that was blocked by OSI-906 (Figure 1C). p53 was also induced to higher levels after CP treatment in S4 cells compared to MHM and co-treatment with OSI-906 reduced p53 accumulation (Figure 1C). IGF-1R/AKT activation coincided with increased *IGF1* and *IGF2* mRNA expression in all the OS cell lines (Figure 1D), suggesting a possible mechanism for how CP activates the pathway. In total, the results indicate that CP activates the IGF-1R/AKT pathway in multiple OS cell lines, and that IGF-1R/AKT pathway activation contributes to the accumulation of p53.

### P53 contributes to IGF-1R/AKT/mTORC1 activation in response to cisplatin

**Figure 2:**
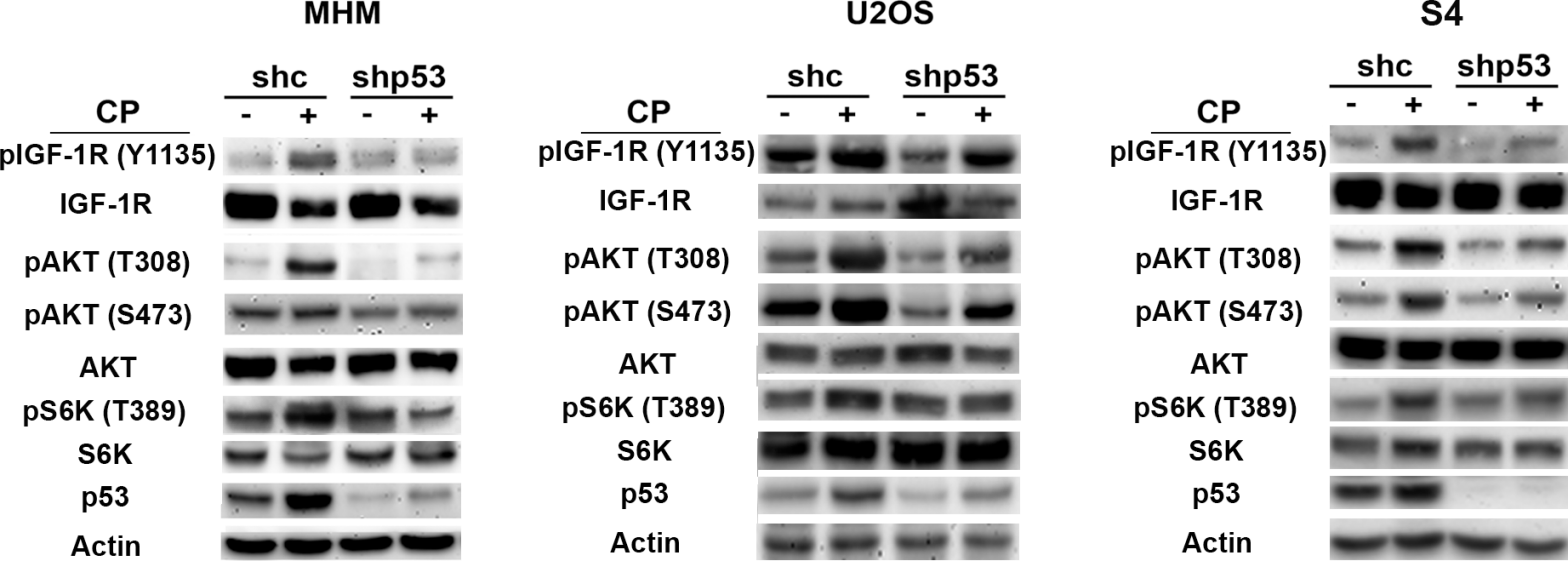
p53 contributes to IGF-1R/AKT/mTORC1 activation MHM, U2OS and S4 cells expressing control shRNA (shc) or p53 shRNA (shp53) were treated with 10 μM CP for 48 hours and expression of the indicated proteins was monitored by immunoblotting.

The fact that OSI-906 reduced p53 levels in CP-treated OS cells supports the idea that IGF-1R/IR/AKT signaling contributes to the accumulation of p53. We wished to ask if p53 might also contribute to IGF-1R/AKT pathway activation. To this end, we treated control and p53 knockdown MHM, U2OS and S4 cells with CP for 48 hours and monitored the levels of pIGF-1R (Y1135), pAKT (S473 and T308) and pS6K (T389, indicative of mTORC1 activity). As shown in Figure 2, pIGF-1R (Y1135), pAKT (S473 and T308) and pS6K (T389) were increased in control cells treated with CP indicating the pathway was activated. However, in the p53 knockdown cells levels of pIGF-1R (Y1135), pAKT (S473 and T308) and pS6K (T389) were induced to a much lower level. This finding supports the idea that p53 contributes to IGF-1R/AKT/mTORC1 pathway activation in CP-treated OS cells.

### IGF-1R inhibition increases p53-dependent apoptotic function

**Figure 3:**
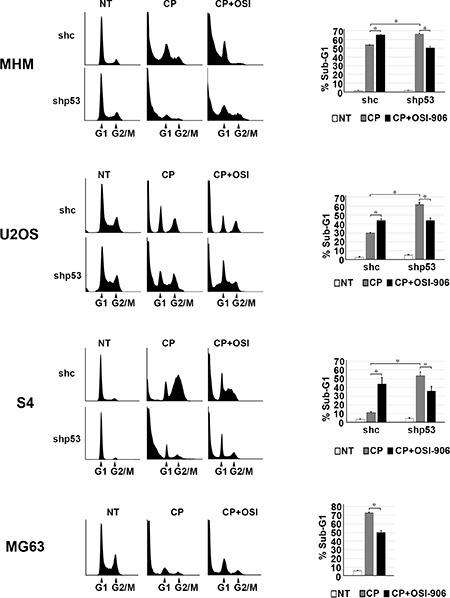
IGF-1R inhibition increases p53 dependent apoptosis and p53 knockdown decreases apoptosis MHM, U2OS and S4 cells expressing control shRNA (shc) or p53 shRNA (shp53) were treated with 10 μM CP and MG63 (p53-null) cells were treated with 5 μM CP alone or in combination with OSI-906 (5 μM) for 48 hours. The cells were then rinsed and re-fed with drug free media and cells were collected after 5 days and analyzed by flow cytometry for cell cycle. Representative cell cycle profile histograms are shown and percentage of cells with Sub-G1 DNA content are plotted. Shown are the mean results of three experiments, *bars*, Standard error (SE). *significance value (*P* < 0.05).

**Figure 4:**
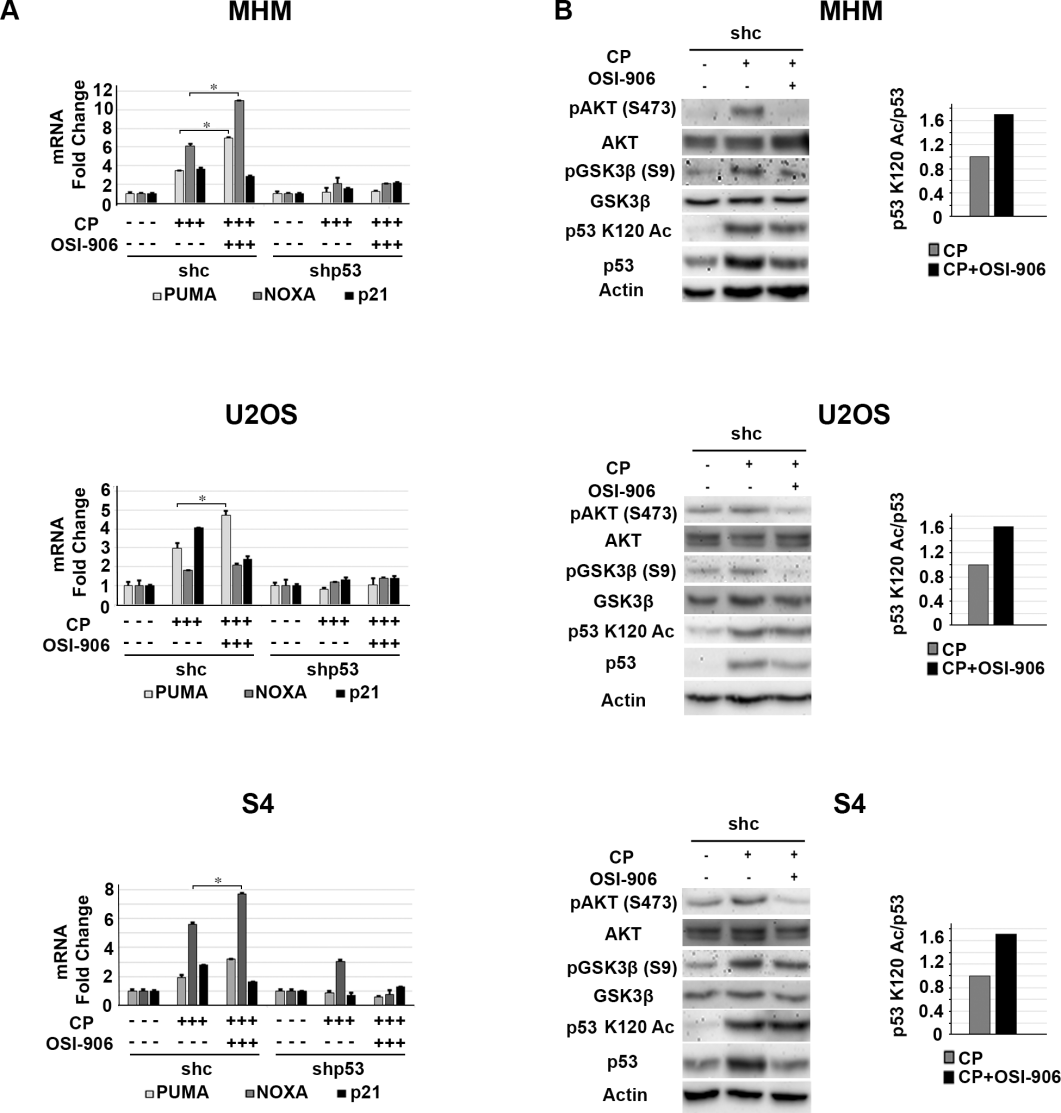
IGF-1R inhibition increases p53 dependent apoptotic gene expression (**A**) MHM, U2OS and S4 cells expressing control shRNA (shc) or p53 shRNA (shp53) were treated with 10 μM CP alone or in combination with OSI-906 (5 μM) for 24 hours and *PUMA, NOXA,* and *P21* mRNA levels were determined by qRT-PCR. The level of each mRNA transcript in untreated cells was considered “1.0” and all the other values were plotted relative to it. Shown are the mean results of three experiments, *bars*, Standard error (SE). *significance value (*P* < 0.05). (**B**) MHM, U2OS and S4 cells expressing control shRNA (shc) were treated with 10 μM CP alone or in combination with OSI-906 (5 μM) for 24 hours and lysates were immunoblotted with indicated antibodies (*Left*). Acetylated p53 (p53 K120 Ac) and total p53 levels were quantified using Image-J software and the relative amount (ratio) of K120-acetylated/total p53 was plotted (*Right*) The p53 K120Ac/p53 ratio in cells treated with CP alone was considered “1.0” and CP plus OSI-906 plotted relative to it.

Results from the previous figures support positive cross-talk between the IGF-1R/AKT/mTORC1 pathway and p53 in CP-treated OS cells. Specifically, the IGF-1R/AKT activation contributes to the accumulation of p53 (Figure 1) and p53 contributes to IGF-1R/AKT/mTORC1 activation (Figure 2). We wished to examine how this cross-talk affects cell fate in response to CP. To this end MHM, U2OS, S4 control and p53 knockdown cells and p53 null MG63 cells were treated with CP alone or CP plus OSI-906 for 48 hours. The cells were then refed with drug-free medium (minus CP and OSI-906). Apoptosis and senescence were monitored 5 days after drug removal and long-term survival (colony formation) was measured 2 weeks after drug removal. Apoptosis was measured by determining the percentage of cells with sub-G1 DNA content. Representative cell cycle profiles (histograms) for each cell line are shown and the percentage of sub-G1 cells plotted in Figure 3. The average percent Sub-G1, G1, S and G2/M populations are shown in Supplementary Table 1. p53 knockdown cells were more susceptible to CP induced apoptosis compared to control cells (Figure 3). These results indicate p53 promotes apoptosis resistance, perhaps by contributing to IGF-1R/AKT/mTORC1 pathway activation. Interestingly, OSI-906 increased apoptosis in control (shc) cells treated with CP, but reduced apoptosis in p53 knockdown (shp53) or p53 null (MG63) cells (see %sub-G1 cells plotted in Figure 3). This result suggested IGF-1R/AKT pathway inhibition can have different effects on apoptosis dependent on p53 expression status. We previously showed AKT inhibitor increased p53-dependent expression of pro-apoptotic genes that contributed to apoptosis [53]. Since AKT is downstream of IGF-1R, we speculated OSI-906 might increase apoptosis in control cells by increasing p53-dependent expression of pro-apoptotic genes. Consistent with this, mRNA levels for the p53 responsive pro-apoptotic genes *PUMA* and *NOXA* were increased in control cells treated with CP and further increased by the combination of CP plus OSI-906, but were not increased in p53 knockdown cells by CP or CP plus OSI-906 (Figure 4A). In contrast, p21 mRNA levels were not further increased in control cells treated with CP plus OSI-906 compared to cells treated with CP alone (Figure 4A). Together, the results of Figure 3 and Figure 4A indicate OSI-906 increases p53-dependent apoptotic gene expression and apoptosis in OS cells that express wild-type p53, but reduces apoptosis in p53 knockdown or p53-null OS cells.

We found it quite interesting that OSI-906 treatment increased p53s apoptotic function despite also causing a reduction in total p53 levels. We wished to examine a possible explanation by which this could occur. The apoptotic function of p53 is regulated in part by post-translational modifications that increase p53 binding to apoptotic gene promoters. Of particular interest is acetylation of p53 at lysine-120 (K120). AKT is activated downstream of IGF-1R in CP-treated cells. GSK3β is a kinase and AKT substrate that can regulate p53 K120 acetylation and activity. Charvet reported GSK3β stimulates p53 apoptotic function (*PUMA* expression) by phosphorylating TIP60 and promoting TIP60-dependent acetylation of p53 at K120 [54]. AKT inhibits GSK3β by phosphorylation at serine-9. Thus we speculated IGF-1R/AKT inhibition might increase p53 apoptotic function by inhibiting AKT and activating GSK3β (reducing the inhibitory phosphorylation of GSK3β at serine 9) and thus promoting K120 acetylation of p53. To examine this possibility we monitored pAKT(S473), pGSK3β (S9), total and K120-acetylated p53 levels in cells treated with CP alone or CP plus OSI-906. As shown in Figure 4B, pAKT (S473) and pGSK3β (S9) levels were increased in CP treated cells and this was blocked by OSI-906. This supports AKT and GSK3β (S9) phosphorylation being downstream of IGF-1R. Levels of p53 acetylated at K120 were increased in response to CP or CP plus OSI-906 (Figure 4B, immunoblots). However, while co-treatment with OSI-906 reduced total p53 levels, K120-acetylated p53 levels were either not reduced or were only minimally affected. This result indicates IGF-1R inhibition induces or maintains p53 K120 acetylation in CP-treated cells. We quantified K120-acetylated and total p53 levels and plotted the ratio (Figure 4B, graphs). The results showed there was an increase in the relative amount of K120-acetylated p53 in cells treated with CP plus OSI-906 compared to cells treated with CP alone. p53 binds DNA as a tetramer/oligomer. We speculate increasing the relative amount of K120-acetylated p53 may result in formation of more K120-acetylated p53 oligomers, or more p53 oligomers in which K120 is acetylated, and that this in turn results in more pro-apoptotic gene activation by p53 (e.g. PUMA) and thus more apoptosis.

### IGF-1R inhibition increases long-term survival in cisplatin-treated cells that lack p53

**Figure 5:**
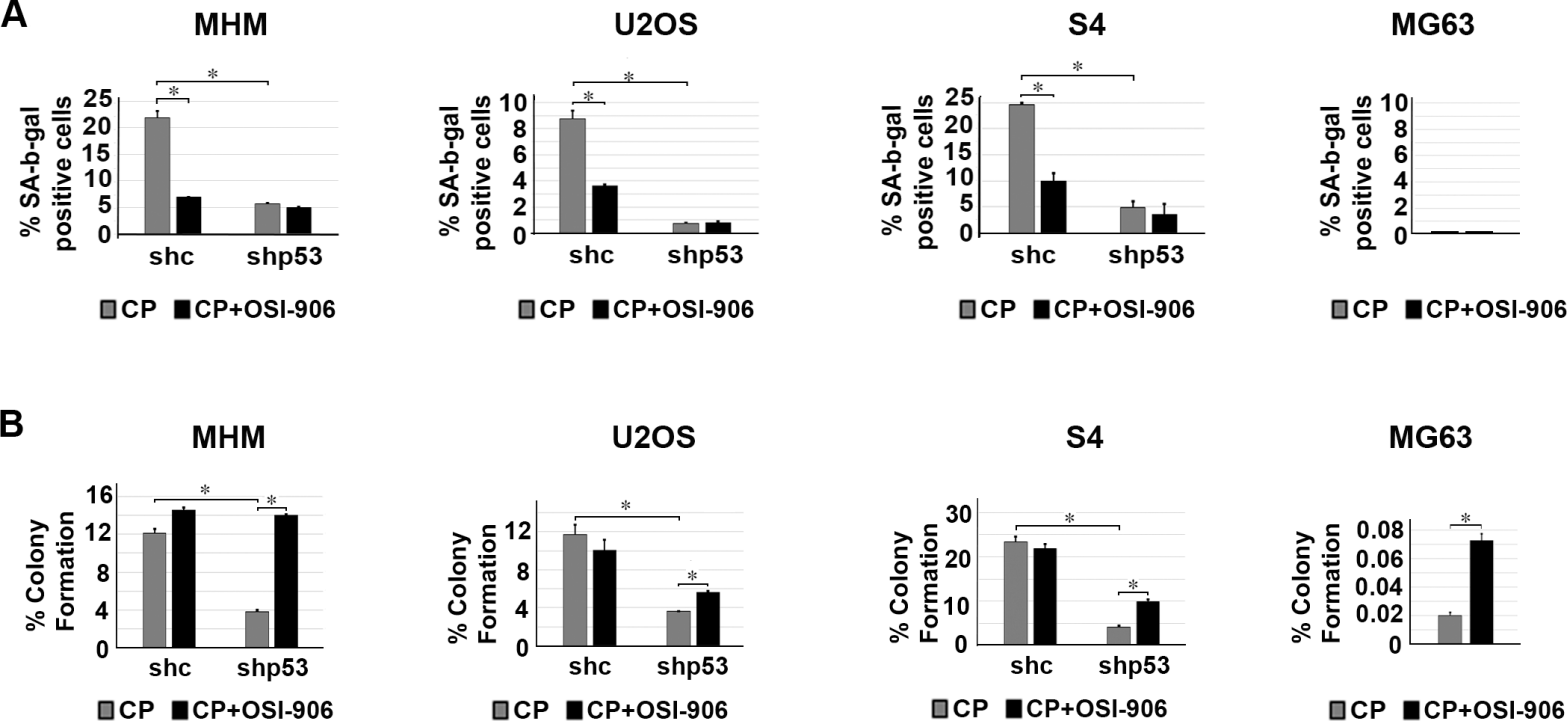
p53 and IGF-1R/AKT activation promotes senescence in CP treated cells and IGF-1R inhibition increases long term survival in cells that lack p53 (**A**) MHM, U2OS and S4 cells expressing control shRNA (shc) or p53 shRNA (shp53) and MG63 (p53-null) cells were treated with 2.5 μM CP alone or in combination with OSI-906 (5 μM) for 48 hours. The cells were then rinsed and re-fed with drug free medium and the percentage of senescent cells (flat and SA-β-gal positive) determined after 5 days. The SA-β-gal positive cells were counted and normalized with plating efficiency. Shown are the mean results of three experiments, *bars,* Standard error (SE). *significance value (*P* < 0.05). (**B**) MHM, U2OS and S4 cells expressing control shRNA (shc) or p53 shRNA (shp53) and MG63 cells were treated with 2.5 μM CP alone or in combination with OSI-906 (5 μM) for 48 hours. The cells were then rinsed and re-fed with drug free media and colonies stained with crystal violet 2–3 weeks later. The colonies were counted and normalized with plating efficiency of untreated cells. Shown are the mean results of three experiments, *bars,* Standard error (SE). *significance value (*P* < 0.05).

Next, we monitored senescence in cells by scoring the percentage of cells that were flat and expressed senescence-associated beta-galactosidase (SA-β-gal). Again, cells were treated with CP alone or CP plus OSI-906 for 48 hours. The cells were then refed with drug-free medium (minus CP and OSI-906) and senescence monitored 5 days later. As shown in Figure 5A, p53 knockdown reduced senescence in MHM, U2OS and S4 cells treated with CP, indicating that p53 promotes or contributes to CP induced senescence. OSI-906 reduced senescence in control cells treated with CP, suggesting that IGR-1R/AKT activation also contributes to senescence. Importantly, we did not observe senescence in p53-null MG63 cells treated with CP alone or CP plus OSI-906, and OSI-906 did not further reduce senescence in cells in which p53 was knocked down. The results indicate that p53 promotes senescence in CP-treated OS cells, and that IGF-1R pathway activation maintains p53 protein levels and thus contributes to p53-dependent senescence.

Next, we monitored long-term survival in OS cells treated with CP or CP plus OSI-906. Similar to Figure 3 and Figure 5A, cells were treated with CP alone or CP plus OSI-906 for 48 hours, then refed with drug-free medium (minus CP and OSI-906). Long term survival (colony formation) was measured 2–3 weeks after drug removal. At least three things became apparent from these studies: First, p53 knockdown reduced colony formation in MHM, U2OS and S4 cells treated with CP (Figure 5B). This indicates that p53 promotes long term survival in CP-treated OS cells. Second, co-treatment with OSI-906 had little to no effect on colony formation in CP-treated control MHM, U2OS and S4 cells despite the ability of OSI-906 to increase apoptosis in these cells (Figure 3). We speculate the failure of OSI-906 to reduce colony formation in CP-treated control cells results from reduced p53 levels and reduced p53-dependent senescence (Figure 5A). Third, OSI-906 increased colony formation in p53 knockdown MHM, U2OS and S4 cells treated with CP and also increased long-term survival in p53-null MG63 (Figure 5B). The ability of OSI-906 to increase colony formation in p53 knockdown (S4 cells) and p53-null cells is consistent with the finding that OSI-906 reduces CP induced apoptosis in these cells (Figure 3).

### P27 expression is maintained in cisplatin treated cells when IGF-1R is inhibited

**Figure 6:**
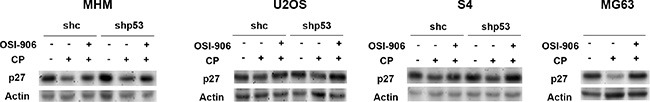
CP reduces p27 levels which is prevented by OSI-906 MHM, U2OS and S4 cells expressing control shRNA (shc) or p53 shRNA (shp53) and MG63 cells were untreated or treated with 10 μM CP alone or in combination with OSI-906 (5 μM) for 24 hours. Lysates were immunoblotted with antibodies against p27 and Actin.

We were intrigued by the finding that OSI-906 reduced apoptosis (Figure 3) and increased long-term survival (Figure 5B) in CP-treated cells that lack p53. Previously, our lab reported that AKT inhibition stabilized p27 in CP-treated OS cells, and p27 then promoted survival by mediating a G1-phase delay/arrest [53]. We therefore speculated IGF-1R inhibition by OSI-906 might stabilize p27 in CP-treated cells that lack p53, and thus promote a p27 dependent G1 arrest/delay that protects cells from CP induced killing. To examine this, p27 levels were first determined in MHM, U2OS, and S4 control and p53 knockdown cells as well as MG63 cells treated with CP alone or CP plus OSI-906. As shown in Figure 6, p27 levels were reduced in CP-treated control and p53 knockdown/null cells, but at least partially restored in cells co-treated with CP plus OSI-906. This is consistent with the idea that CP reduces p27 levels in a manner that is IGF-1R dependent.

### Maintaining p27 contributes to apoptosis resistance and long-term survival in cisplatin-treated cells that lack p53

**Figure 7:**
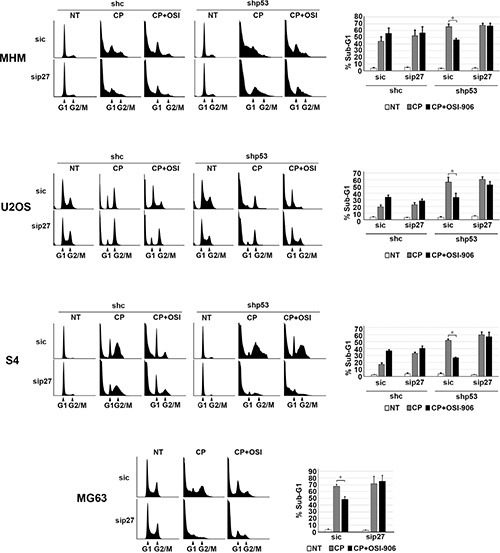
Maintaining p27 levels contributes to reduced apoptosis in p53 knockdown/null cells treated with CP plus OSI-906 MHM, U2OS and S4 cells expressing control shRNA (shc) or p53 shRNA (shp53) and MG63 (p53-null) cells were transfected with control siRNA (sic) and p27 siRNA (sip27) and treated with 10 μM CP alone or in combination with OSI-906 (5 μM) for 48 hours. The cells were then rinsed and re-fed with drug free medium and cells were collected 5 days and analyzed by flow cytometry for cell cycle. Representative cell cycle profile histograms are shown and percentage of cells with Sub-G1 DNA content are plotted. Shown are the mean results of three experiments, *bars*, Standard error (SE). *significance value (*P* < 0.05).

**Figure 8:**
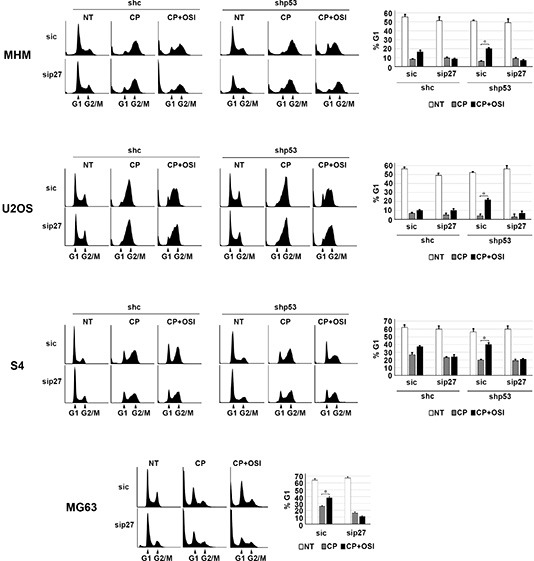
OSI-906 causes a G1 arrest/delay in CP treated cells MHM, U2OS, and S4 cells expressing control shRNA (shc) or p53 shRNA (shp53) and MG63 (p53-null) cells were transfected with control siRNA (sic) and p27 siRNA (sip27) and treated with 2.5 μM CP alone or in combination with OSI-906 (5 μM) for 48 hours. Representative cell cycle profile histograms at the time of harvest are shown (*Left*) and the percentage of G1-phase cells plotted +/− SE from 3 experiments (*Right*).

**Figure 9:**
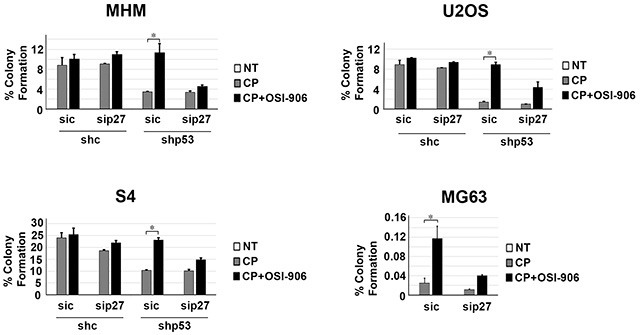
Maintaining p27 levels contributes to increased long term survival in p53 knockdown/null cells treated with CP plus OSI-906 MHM, U2OS, and S4 cells expressing control shRNA (shc) or p53 shRNA (shp53) and MG63 (p53-null) cells were transfected with control siRNA (sic) and p27 siRNA (sip27) and treated with 2.5 μM CP alone or in combination with OSI-906 (5 μM) for 48 hours. The cells were then rinsed and re-fed with drug free medium and colonies stained with crystal violet 2–3 weeks later. The colonies were counted and normalized with plating efficiency of untreated cells. Plotted are the mean results of three experiments, *bars,* Standard error (SE). *significance value (*P* < 0.05).

Next, we wished to ask if maintaining p27 levels contributes to the reduction in apoptosis and increased long-term survival in p53 knockdown/null cells treated with CP plus OSI-906. To test this, control and p53 knockdown MHM, U2OS, and S4 cells as well as MG63 cells were transfected with control (non-target) siRNA or p27 siRNA. The cells were then treated with CP alone or CP plus OSI-906 for 48 hours, and then refed with drug-free medium (minus CP and OSI-906). Apoptosis was monitored 5 days later by determining the percentage of cells with sub-G1 DNA content. Immunoblotting confirmed that p27 was efficiently knocked down (Supplementary Figure 2). As shown in Figure 7, in control MHM, U2OS and S4 cells p27 knockdown had little effect on CP-induced apoptosis (representative histograms at the time of cell harvest and the % cells with sub-G1 DNA content plotted in Figure 7). The average percent Sub-G1, G1, S and G2/M populations for each cell line in this experiment are shown in Supplementary Table 2. However, in the p53 knockdown cells and in null (MG63) cells co-treatment with OSI-906 reduced apoptosis and this effect was abrogated by knockdown of p27. This indicates the ability of OSI-906 to reduce CP-induced apoptosis in these cells is p27 dependent. We next asked if increased colony formation in p53-null/knockdown cells co-treated with CP plus OSI-906 also required p27, and if it was associated with a p27-dependent G1 arrest/delay. First, control and p53 knockdown MHM, U2OS, and S4 cells as well as MG63 cells were transfected with control (non-target) siRNA or p27 siRNA, and subsequently treated with CP alone or CP plus OSI-906 for 48 hours. The distribution of cells in G1, S, and G2/M phases were then quantified. In all cases, treatment with CP alone caused an accumulation of cells in S and G2/M phase with a corresponding reduction in G1-phase cells. However, co-treatment with OSI-906 increased the percentage of cells in G1-phase (Figure 8, compare cell cycle profile in cells treated with CP alone vs CP+OSI; also compare the percentage of G1-phase cells from multiple experiments graphed in Figure 8). This was true in both control and p53-knockdown/null cells though it was more evident in p53-knockdown/null cells. The percentage of cells in each cells in each cell cycle phase was quantified in these experiments and is presented in Supplementary Table 3. Most importantly, the increase in G1-phase cells upon CP plus OSI-906 treatment was largely absent in cells where p27 was knocked down (Figure 8). These results indicate co-treatment with OSI-906 causes an accumulation of G1-phase cells (G1 arrest/delay) that is largely p27-dependent. Finally, we measured colony formation in control (sic) and p27 knockdown cells treated with CP alone or CP plus OSI-906. As shown in Figure 9, p27 knockdown had little effect on colony formation in control MHM, U2OS, or S4 cells treated with CP, and OSI-906 also had minimal effect on colony formation in these cells. However, OSI-906 increased colony formation in the p53 knockdown and p53-null (MG63) cells, and these effects were reduced/abrogated by knockdown of p27 (Figure 9). This indicates the ability of OSI-906 to increase long term survival in CP-treated cells that lack p53 is p27-dependent. In sum, results from Figures 7–9 support the idea that IGF-1R inhibition by OSI-906 promotes a p27 dependent G1 arrest/delay that protects p53-null/knockdown cells from CP induced apoptosis and increases long-term survival.

## DISCUSSION

Cancer responsiveness to chemotherapy is controlled in part by the relative balance of activated tumor suppressors that inhibit proliferation or induce death, and survival signaling pathways that maintain proliferative capacity and block apoptosis. The IGF-1R/AKT pathway can be activated in response to chemotherapy and can increase survival and chemotherapy resistance [1–5, 55, 56]. In contrast, the tumor suppressors p53 and p27 can inhibit cancer cell proliferation by inducing cell cycle arrest, senescence and, in the case of p53, apoptosis [7, 9, 22, 24, 25, 31]. There is abundant crosstalk between the IGF-1R/AKT/mTORC1 pathway and p53 and p27 [35, 37, 38, 42, 57]. However, the impact of this crosstalk on DNA damage/therapy response in cancer and chemotherapy resistance is largely unknown.

**Figure 10:**
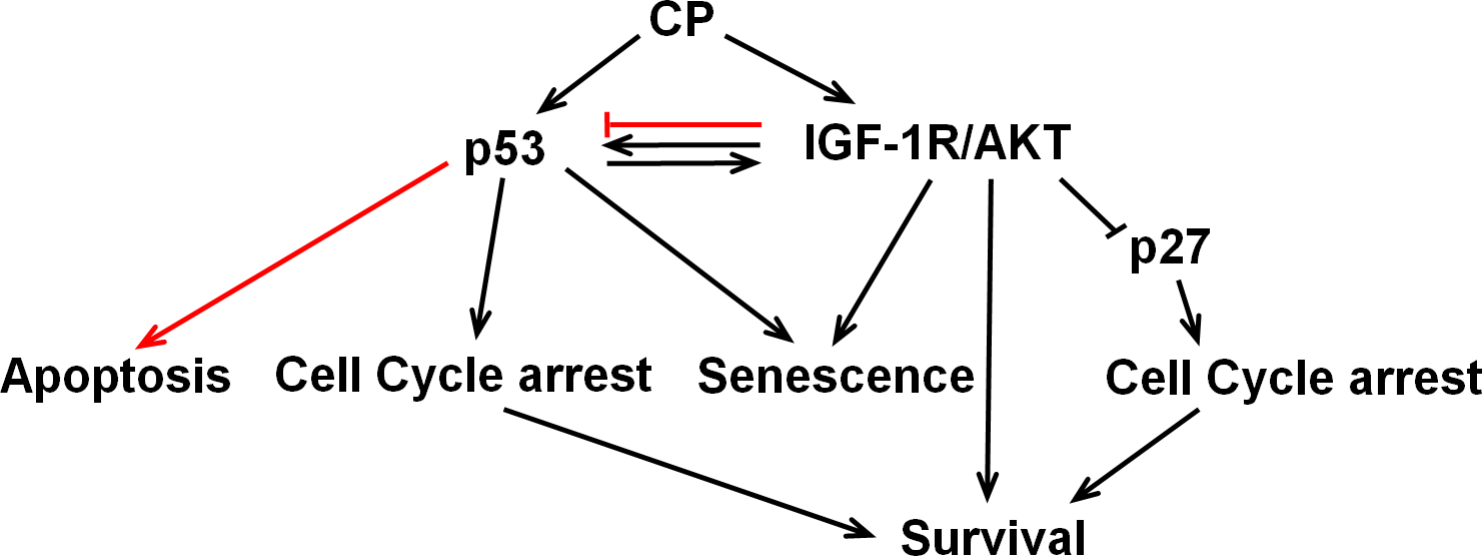
Working model CP activates p53 and the IGF-1R/AKT pathway. p53 and IGF-1R/AKT have positive crosstalk in CP-treated OS cells. IGF-1R/AKT activation contributes to the accumulation of p53 and p53 contributes to the IGF-1R/AKT activation. IGF-1R/AKT pathway activation maintains p53 protein levels and p53-dependent senescence, but inhibits p53s apoptotic function (red line). P53 contributes to IGF-1R/AKT activation and IGF-1R/AKT dependent survival. P53 may also increase survival by inducing cell cycle arrests that allow DNA repair. IGF-1R/IR inhibition had no effect on long-term survival in p53-expressing cells due to opposing effects of reducing p53-dependent senescence while increasing p53-dependent apoptosis. In contrast, IGF-1R/IR inhibition in CP-treated cells that lack p53 increased long term survival by stabilizing p27.

In the current report we examined how crosstalk between IGF-1R/AKT/mTORC1 pathway and the p53 and p27 affects cancer cell responses to the standard chemotherapy agent cisplatin (CP) (Figure 10). In p53 wild-type cells treated with CP, IGF-1R inhibition reduced p53 protein levels and p53-dependent senescence but increased p53-mediated apoptosis, and had no effect on long-term survival. In contrast, in p53-null or p53-knockdown cells, IGF-1R inhibition reduced apoptosis after CP treatment and increased long-term survival. These effects were due at least in part to p27 since IGF-1R inhibition stabilized p27 in CP-treated cells and p27 depletion restored apoptosis sensitivity and reduced long-term survival. These results demonstrate that 1) p53 status (expression) can determine the effect of IGF-1R inhibition on cancer cell responses to CP, and 2) crosstalk between the IGF-1R/AKT/mTORC1 pathway and p53 and p27 can reduce cancer cell responsiveness to chemotherapy (CP), which could ultimately limit the effectiveness of IGF-1R pathway inhibitors in the clinic.

The IGF-1R/AKT/mTORC1 pathway is aberrantly activated in multiple cancers and there is abundant literature that this pathway contributes to apoptosis resistance and survival [2–6, 56]. Most studies suggest that p53 can inhibit the IGF-1R/AKT/mTORC1 pathway and conversely that the IGF-1R/AKT/mTORC1 activation inhibits p53 [35, 37, 42, 57, 58]. In the current report, CP activated the IGF-1R/AKT/mTORC1 pathway in multiple OS cell lines. We found this coincided with increased IGF1 and IGF2 gene expression, suggesting CP may activate the pathway in part by increasing expression of IGF1/2. p53 knockdown by shRNA reduced IGF-1R/AKT/mTORC1 activation, indicating that p53 contributes to IGF-1R/AKT/mTORC1 activation in CP-treated OS cells. We considered the possibility that p53 might increase IGF-1R/AKT/mTORC1 activation by increasing expression of IGF1 and/or IGF2. However, in our ongoing studies (not shown) we found IGF1 and IGF2 mRNAs were induced to higher levels after CP treatment in cells in which p53 was knocked down compared to control cells. This finding is consistent with reports that p53 can repress *IGF1* gene expression and indicates p53 does not activate the IGF-1R/AKT/mTORC1 pathway by increasing expression of IGF1 or IGF2 [37, 42]. We also considered p53 might increase AKT activation in CP-treated cells by inhibiting mTORC1 mediated activation of S6K and thus relieving the feedback inhibition of AKT that is mediated by pS6K [12, 13]. However, pS6K (indicative of mTORC1 activity) were not lower in control cells compared to p53 knockdown OS cells treated with CP (Figure 2). This suggests p53 does not contribute to IGF-1R/AKT/mTORC1 activation by inhibiting the mTORC1-S6K signaling axis. Insulin Growth Factor Binding Protein-3 (IGF-BP3) is a p53-responsive factor that can bind and sequester IGF1 to reduce IGF-1R pathway signaling [46, 47, 59, 60]. However, recent reports showed that IGFBP-3 can also potentiate the mitogenic effects of IGF1, perhaps by blocking IGF1 degradation [47, 61, 62]. One possibility is that p53 contributes to IGF-1R/AKT/mTORC1 activation by inducing IGFBP-3, which then potentiates IGF1-dependent activation of this pathway.

IGF-1R/AKT/mTORC1 pathway activation can affect the levels and activity of p53 in diverse ways. For example, Mayo and colleagues reported that IGF1 promoted the activation of AKT, and activated AKT then phosphorylated MDM2 and stimulated MDM2-dependent p53 degradation [48]. Similarly, Xiong et al. reported that IGF-1R inhibition stabilized the p53 protein in MEFs and therapy treated cancer cells, potentially by inhibiting the AKT and MDM2-dependent degradation pathway described by Mayo and colleagues [51]. In contrast to these studies are the findings of Blattner and colleagues in which it was reported that AKT activated by ionizing radiation (IR) promoted the stabilization of p53 [50]. Still other studies have suggested that mTORC1 activated downstream of IGF-1R can promote p53 protein translation/synthesis [51, 52]. In the current report we found that IGF-1R inhibition reduced the accumulation of p53 in CP-treated cells. In continuing studies we found AKT and mTORC1/2 inhibitors also reduced p53 accumulation in CP-treated cells (Supplementary Figure 3A and 3B), and that the mTORC1 inhibitor rapamycin reduced p53 protein synthesis and caused a slight reduction in p53 mRNA levels (Supplementary Figure 3C). The results suggest IGF-1R/AKT/mTORC1 activation promotes/maintains p53 protein levels at least in part through a combination of increasing p53 protein synthesis and maintaining p53 mRNA.

p53 levels and activity increase in response to DNA damaging stress. The effect of increasing p53 is to induce either apoptosis or transient/permanent cell cycle arrest. Apoptosis or permanent cell cycle arrest (senescence) are bona-fide tumor suppressor mechanisms through which p53 inhibits cancer cell survival. In contrast, transient cell cycle arrests induced by p53 can increase survival by allowing cells time to repair their DNA before proceeding with cell division. An important yet unresolved question is how the choice of response to p53 (apoptosis vs arrest) is regulated. Our data suggest IGF-1R/AKT signaling can regulate the choice of response to p53. The IGF-1R/AKT pathway was activated in CP-treated OS cells. p53 knockdown increased apoptosis and reduced long-term survival in OS cells treated with CP, indicating that p53 promotes survival in OS cells in response to CP. This could result from transient arrests mediated by p53 that allow DNA repair. However, we also found the IGF-1R/AKT pathway was less activated in p53 knockdown cells, and we therefore speculate p53 can also reduce apoptosis and increase survival by maintaining or contributing to IGF-1R/AKT activation. p53 was induced to a lower level in cells treated with CP plus IGF-1R/IR inhibitor (OSI-906) compared to cells treated with CP alone. However, despite p53 protein levels being lower, the apoptotic function of p53 appeared to increase. This was evidenced by the finding that IGF-1R inhibition increased p53-dependent apoptotic gene expression (*PUMA, NOXA*) and p53-dependent death after CP. The results indicate IGF-1R signaling maintains p53 protein levels but inhibits its apoptotic function. This raises the question how IGF-1R inhibition increases p53 apoptotic function in response to CP. The apoptotic function of p53 is regulated in part by post-translational modifications that increases p53 binding to apoptotic gene promotors, including acetylation of p53 at lysine-120 (K120). Charvet reported GSK3β stimulates p53 apoptotic function (*PUMA* expression) by phosphorylating TIP60 and promoting TIP60-dependent acetylation of p53 at K120 [54]. AKT inhibits GSK3β by phosphorylation at serine-9 (S9), and we therefore speculated that IGF-1R/AKT inhibition by OSI-906 might increase p53 apoptotic function by increasing K120 acetylation of p53. Total and K120-acetylated p53 levels increased in CP-treated cells. However, while IGF-1R inhibition reduced total p53 levels in response to CP and also reduced pAKT (S473) and pGSK3β (S9) levels, it had little effect on levels of K120-acetylated p53. Protein quantification showed there was an increase in the relative amount of K120-acetylated p53 in cells treated with CP plus OSI-906 compared to cells treated with CP alone. Given that p53 binds DNA as a tetramer/oligomer, we speculate increasing the relative amount of K120-acetylated p53 may result in formation of more K120-acetylated p53 oligomers, or more p53 oligomers in which K120 is acetylated, and that this in turn results in more pro-apoptotic gene activation by p53 (e.g. PUMA) and thus more apoptosis.

Multiple inhibitors of the IGF-1R pathway have been developed and are in various phases of clinical testing [14, 15, 17, 63–65]. Our results suggest the effectiveness of these inhibitors may be dependent, in part, on p53-status. In control cells that express p53, IGF-1R inhibition reduced p53-dependent senescence in response to CP but increased p53-dependent apoptosis. In long-term survival assays (colony formation) IGF-1R inhibition had no effect. We suspect the failure of IGF-1R inhibition to reduce long term survival in these cells was due to the opposing influences of reducing senescence while increasing apoptosis. In contrast, IGF-1R inhibition increased colony formation in p53 knockdown cells and p53-null cells treated with CP. We found this effect was dependent on p27, a cyclin-cdk inhibitor that like p21 can arrest cells in G1-phase [9]. AKT activated downstream of IGF-1R can phosphorylate p27, leading to its degradation and cytoplasmic sequestration [7, 8]. We previously showed AKT inhibitors stabilized p27 in CP-treated cells, which then led to a protective G1-arrest in cells that lack p53 [53]. In the current report, p27 levels were decreased in CP-treated OS cells and this was blocked by IGF-1R inhibition. This result indicates the reduction in p27 required IGF-1R and most likely resulted from AKT-mediated p27 degradation. We found that IGF-1R inhibition maintained p27 levels in CP-treated cells and induced a G1 arrest/delay that was p27-dependent. Further, IGF-1R inhibition reduced apoptosis and increased colony formation in p53 knockdown/null cells, and these effects were reversed by p27 knockdown. These results indicate IGF-1R inhibition reduces apoptosis and increases long-term survival in p53 knockdown/null OS cells in a p27-dependent manner. The most likely scenario is that IGF-1R inhibition blocks AKT-dependent degradation of p27, and stabilized p27 then mediates cytoprotective arrest or delay in G1-phase.

Finally, we note that our findings have potential implications for the clinical use of the IGF-1R pathway inhibitors to enhance chemotherapy responses. Multiple IGF-1R/IR pathway inhibitors have been developed as potential therapeutics. However, while these inhibitors have shown promise in pre-clinical studies when combined with chemotherapy, they have largely failed to increase long-term patient survival. Based on our results we predict IGF-1R/IR inhibitors may fail to enhance therapy responses in p53 wild-type cancers due to the potentially opposing effects of reducing p53-dependent senescence while increasing p53-dependent apoptosis. Further, we predict IGF-1R/IR inhibitors could reduce the effectiveness of chemotherapy against cancers that lack wild-type p53 expression by stabilizing p27 and thus causing p27-dependent cancer cell survival.

## MATERIALS AND METHODS

### Cell lines and reagents

SJSA1, U2OS, MG63 osteosarcoma cells were obtained from ATCC. MHM cells were kindly provided by Dr.Ola Myklebost, Norwegian Radium Hospital. MHM and SJSA1 cells were grown in RPMI medium and U2OS and MG63 in DMEM medium supplemented with 10% fetal bovine serum (FBS), penicillin (100 U/mL) and streptomycin (100 μg/mL). In order to obtain primary OS cells, surgically resected tumor samples were placed in a cell culture dish containing phosphate-buffered saline (PBS), penicillin (100 U/mL) and streptomycin (100 μg/mL), and rinsed with PBS containing penicillin and streptomycin 2–3 times. The tissues were then placed in a new dish and minced into pieces and digestive solution (0.2% Dispase II and 0.1% Collagenase A in PBS or DMEM) was then added. The tissue bits/digestive solution mix was transferred to a conical tube and placed at 37°C for 60 minutes with occasional mixing. The cells were collected by brief centrifugation. The digestive solution was discarded and cells were maintained and grown in complete DMEM medium supplemented with non-essential amino acids and sodium pyruvate. Cells were plated 48 hours before being treated with Cisplatin (Bedford Laboratory) at the indicated concentrations. OSI-906, MK2206, rapamycin and AZD8055 were obtained from Selleck chemicals.

### Immunoblotting

Whole cell extracts were prepared by resuspending cell pellets in lysis buffer (150 mM NaCl, 5 mM EDTA, 0.5% Nonidet P-40, 50 mM Tris, pH 7.5), resolved by SDS-PAGE, and transferred to polyvinylidene difluoride membranes (NEN Life Science Products). Antibodies to pIGF-1R (Y1135), IGF-1R, pAKT (S473), pAKT (T308), AKT (C67E7), p-p70S6K (T389), p70S6K (49D7), pGSK3β (S9) and GSK3β (27C10) were from Cell Signaling. Antibodies to β-Actin (C4) and p53 (Ab-6) were from Santa Cruz Biotechnology and acetyl-p53 (Lys120) (ABE286) was from EMD Millipore. Primary antibodies were detected with goat anti-mouse (Pierce) or goat anti-rabbit (Life Technologies) secondary antibodies conjugated to horseradish peroxidase, using Clarity chemiluminescence (Bio-Rad).

### mRNA isolation and analysis

Total RNA were prepared using Total RNA mini kit (IBI Scientific, IA); the first cDNA strand was synthesized using high capacity cDNA Reverse Transcription kit (Applied Biosystems, CA) following manufacturer's protocol. Quantitative real-time PCR (qRT-PCR) was performed to measure mRNA levels for *IGF1*, *IGF2*, *PUMA*, *NOXA*, *P21* and β-Actin. The quantitative real-time PCR reaction was run in a 7300 Real Time PCR System (Applied Biosystems, Foster, CA) using EvaGreen qPCR master mix (Midwest Scientific) following manufacturer's instructions. Thermocycling was done in a final volume of 20 μL containing 2 μL of cDNA and 400 nmol/L of primers (Primers are listed in Supplementary Table 4). All samples were amplified in triplicate using the following cycle scheme: 95°C for 2 minutes, 40 cycles of 95°C for 15 seconds and 55°C for 60 seconds. Fluorescence was measured in every cycle and mRNA levels were normalized using the Actin values in all samples. A single peak was obtained for targets, supporting the specificity of the reaction. Semi-quantitative real time PCR was performed to measure mRNA levels for p53 and β-actin. PCR products were loaded onto 2% ethidium bromide stained agarose gel. Images were acquired and quantification of the bands were performed on Image J. mRNA levels were normalized using Actin levels. Primers used for qRT-PCR are listed in Supplementary Table 4.

### siRNA and shRNA mediated knockdown

p53 siRNA (On-target plus smart pool) and control RNAi (On-target plus siControl non-targeting pool) were purchased from Dharmacon and were transfected following manufacturer's guidelines using DhramaFECT I reagent. U2OS with shRNA mediated stable p53 knockdown and the corresponding control cells (shc) were previously described [53]. MHM and S4 control (shc) and stable p53 knockdown (shp53) cells were also previously described [66]. In these cells, p53 is knocked down in a doxycycline-inducible manner, and therefore control (shc) and p53 knockdown (shp53) cells were grown in medium containing 100 ng/ml doxycycline.

### Flow cytometry analysis

For apoptosis (% sub-G1 cells) and cell cycle analysis, cells were harvested and fixed in 25% ethanol overnight. The cells were then stained with propidium iodide (25 μg/ml; Calbiochem). Flow cytometry analysis was performed on Gallios^™^ flow cytometer (Beckman Coulter) and analyzed with FlowJo 8.7 (Treestar, Inc). For each sample, 10,000 events were collected.

### Clonogenic assay

Cells were plated 48 hours before being untreated or treated with CP alone or plus OSI-906 in appropriate dilutions to form 50–100 colonies. After 48 hours of treatment cells were rinsed 3 times with PBS and complete media was added. After 2–3 weeks colonies were fixed with formaldehyde and stained with 0.05% crystal violet. The colonies were counted and normalized with the plating efficiency of untreated cells.

### Cell senescence assay

Cells were plated 48 hours before being untreated or treated with CP alone or plus OSI906. After 48 hours of treatment cells were rinsed 3 times with PBS and complete media was added. After 5 days cells were fixed and stained with senescence β-galactosidase staining kit from Cell Signaling following manufacturer's protocol. Flat and β-galactosidase positive cells were counted and normalized with the plating efficiency of untreated cells.

## SUPPLEMENTARY MATERIALS FIGURES AND TABLES


