# Supplementary material for: Crosstalk between the IGF-1R/AKT/mTORC1 pathway and the tumor suppressors p53 and p27 determines cisplatin sensitivity and limits the effectiveness of an IGF-1R pathway inhibitor
Source: Oncotarget. 2016 Mar 30;7(19):27511–26. doi: 10.18632/oncotarget.8484 (PMC5053668; doi:10.18632/oncotarget.8484)
Supplement: Supplementary file 1 [file oncotarget-07-27511-s001.pdf]

Crosstalk between the IGF-1R/AKT/mTORC1 pathway and the tumor suppressors p53 and p27 determines cisplatin sensitivity and limits the effectiveness of an IGF-1R pathway inhibitor

Supplementary Materials

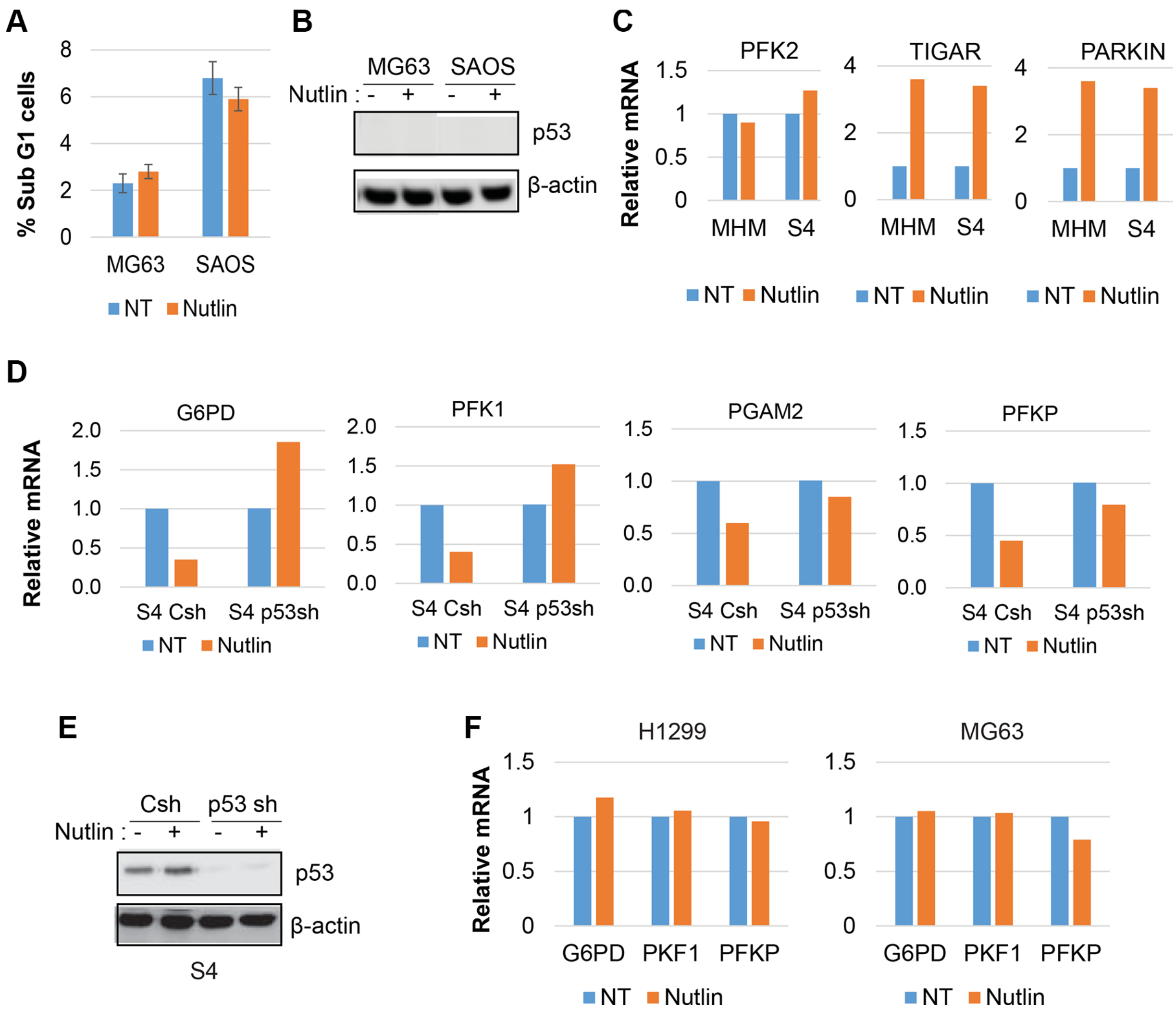

Supplementary Figure S1: Nutlin-induced apoptosis and downregulation of glycolytic genes is dependent on p53. (**A**) p53-null MG63 and SAOS cells were treated with vehicle (NT) or Nutlin (10 μM) for 72 hours and analyzed for sub-G1 cells. (**B**) The cells were treated with vehicle (NT) or Nutlin for 24 hours and lysates were immunoblotted for p53 and β-actin. (**C**) MHM and S4 cells were treated with vehicle or Nutlin for 24 hours and mRNA was analyzed for the indicated genes. (**D**) S4 cells with stable expression of control shRNA or p53 shRNA were treated with vehicle (NT) or Nutlin for 24 hours and mRNA was quantitatively analyzed for the indicated gene expression. (**E**) Whole cell lysates were immunoblotted for p53 and β-actin. (**F**) p53 null H1299 and MG63 treated with vehicle or Nutlin for 24 hours and mRNA was analyzed for the indicated genes.

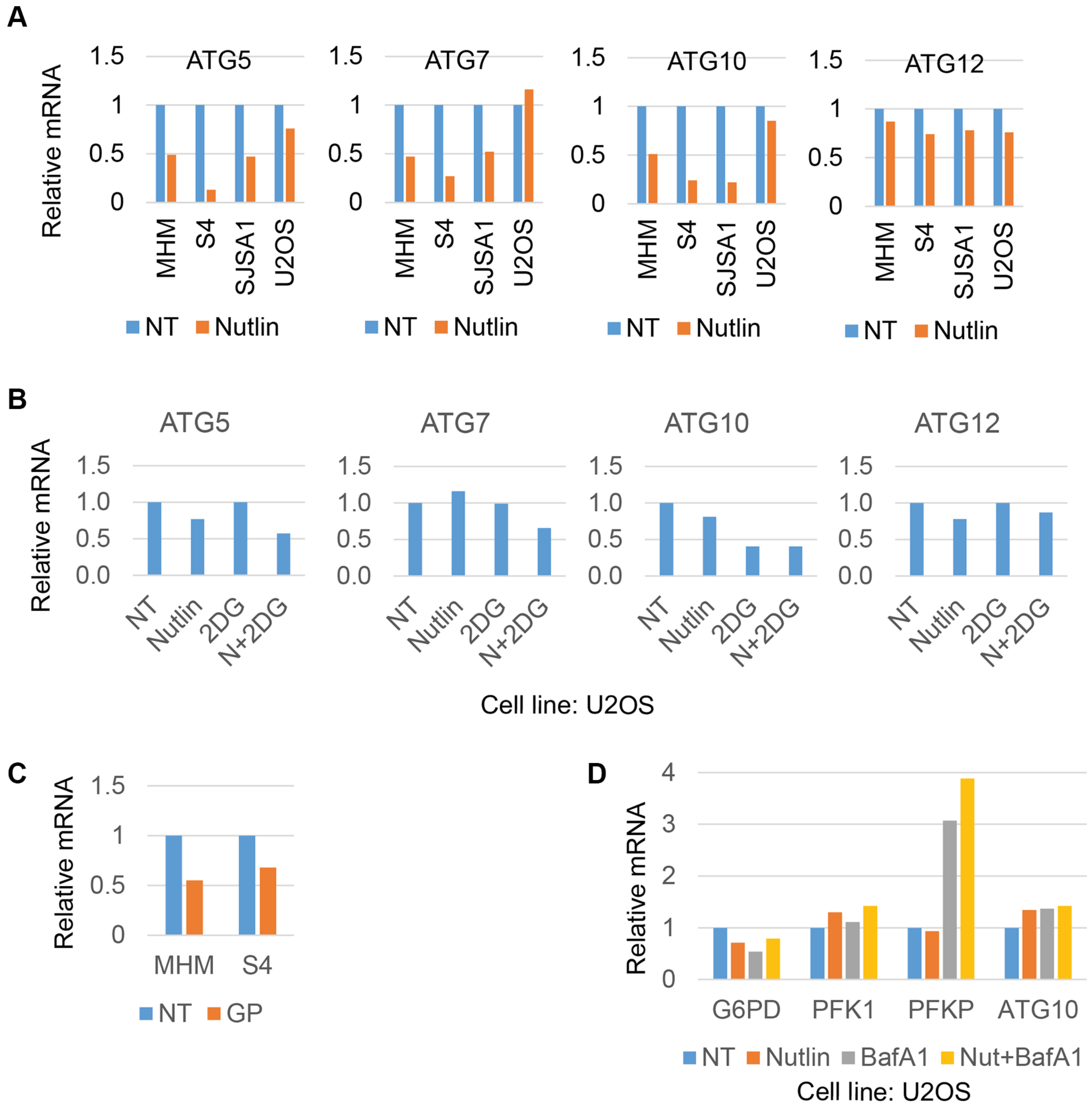

Supplementary Figure S2: Nutlin downregulates ATG genes by inhibition of glycolysis in sensitive cells but not in resistant cells. (**A**) The indicated cell lines were treated with vehicle (NT) or Nutlin for 24 hours and mRNA was quantitatively analyzed for the indicated gene expression. (**B**) U2OS cells were treated with vehicle (NT) or Nutlin in the presence or absence of 2-DG (0.1 mM) for 24 hours and mRNA was quantitatively analyzed for the indicated gene expression. (**C**) MHM and S4 cells were grown in 10 mM glucose (NT) or 0.1 mM glucose (GP) for 12 hours and mRNA was quantitatively analyzed for ATG10 gene expression. (**D**) U2OS cells were treated with vehicle, Nutlin, Bafilomycin A1, or Nutlin plus Bafilomycin A1 for 24 hours and mRNA was quantitatively analyzed for the indicated gene expression.

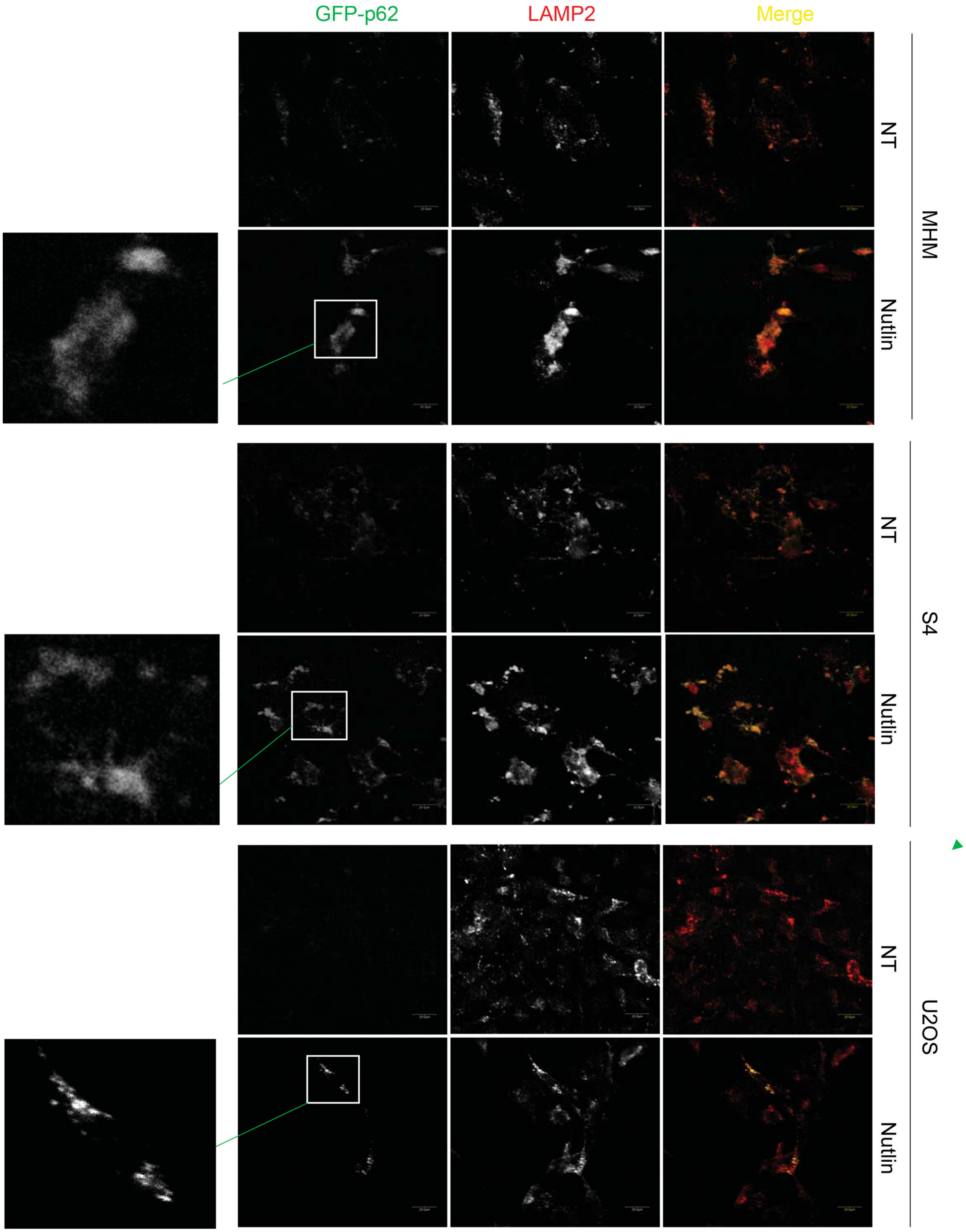

Supplementary Figure S3: Nutlin induces formation of p62 aggregates in sensitive cells. (**A**) MHM, S4, and U2OS cells were transfected with GFP-p62 and then treated with vehicle (NT) or nutlin for 24 hours. The cells were then immunostained for LAMP2 (red) and scanned with a confocal microscope. Note that in MHM and S4 cells Nultin treatment increased fluorescence intensity of GFP-p62 which form patches which are different from U2OS cells in which GFP-p62 show punctate staining which colocalize with LAMP2 labeled lysosomes.

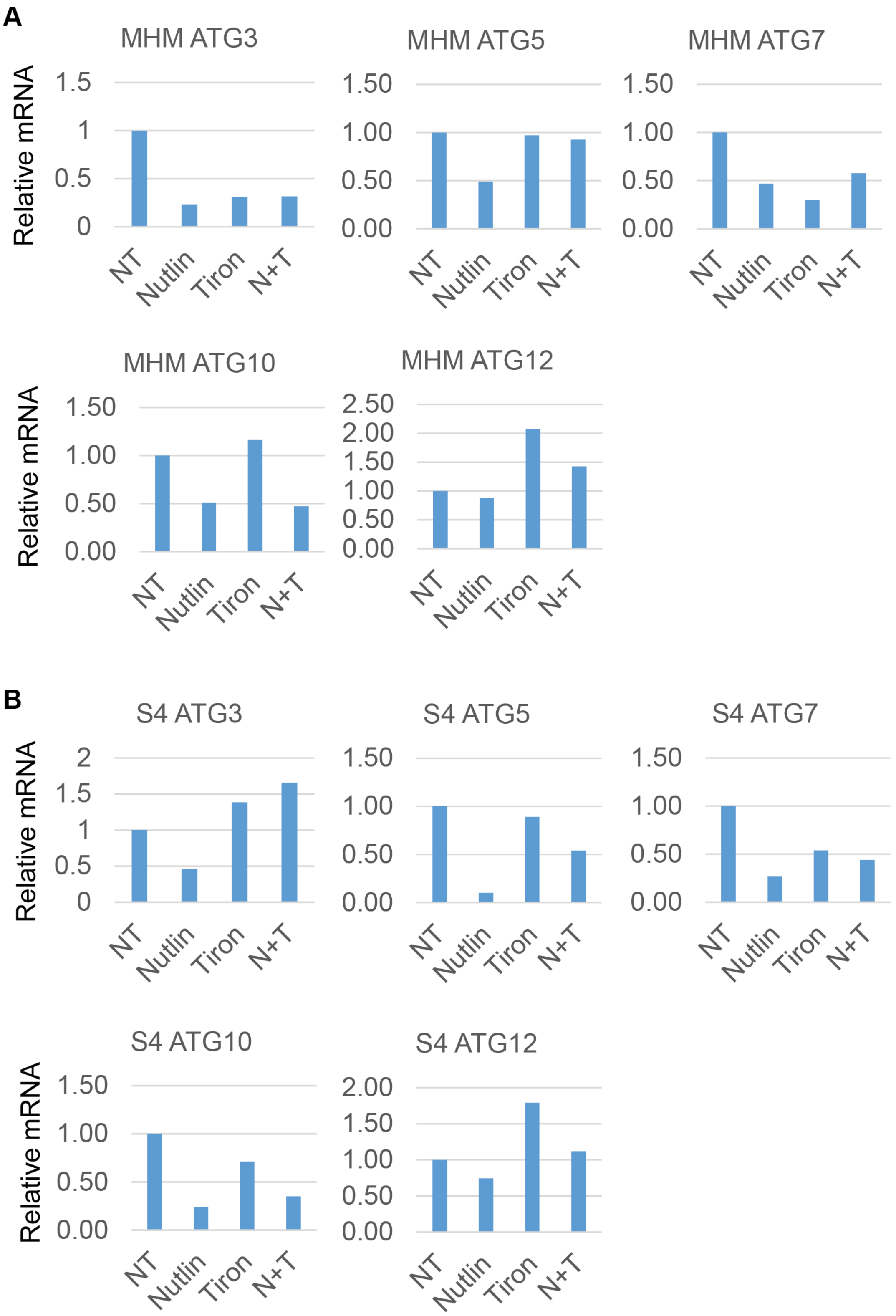

Supplementary Figure S4: Superoxide scavenging maintains ATG genes. MHM (**A**) and S4 (**B**) cells were treated with vehicle (NT) or Nutlin for 24 hours. mRNA was quantitatively analyzed for the indicated gene expression.

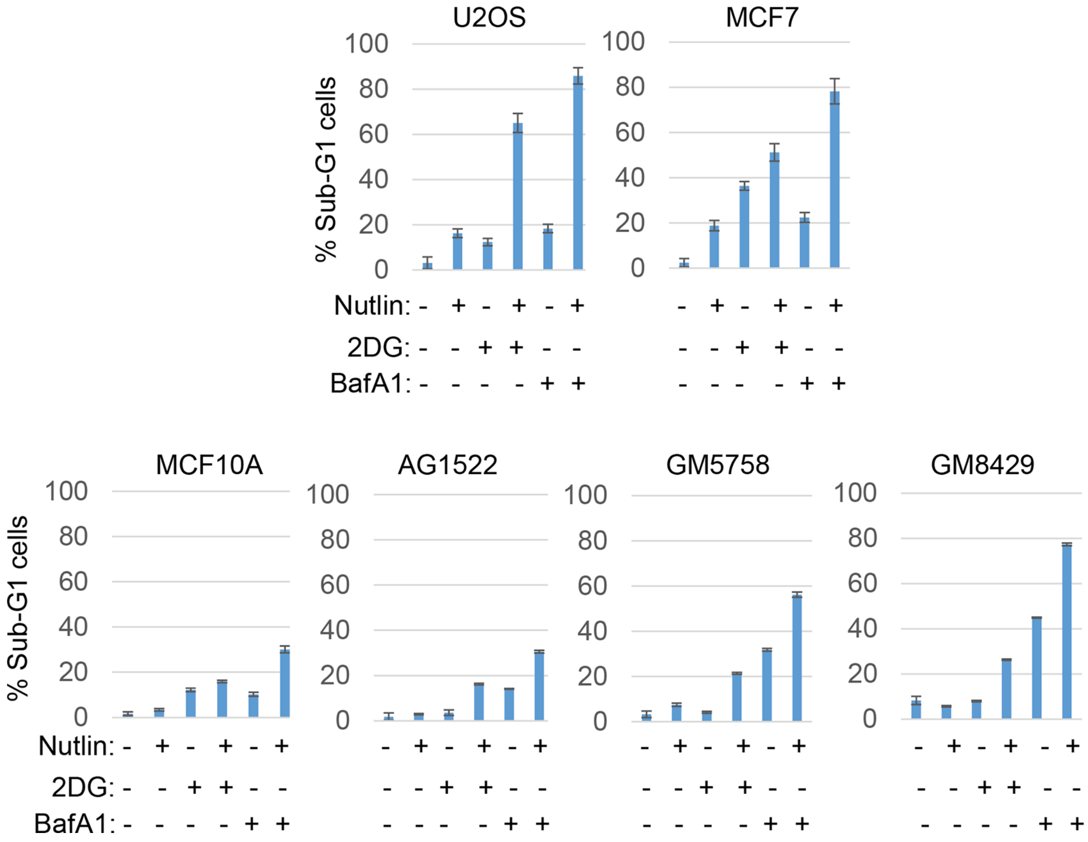

Supplementary Figure S5: Non-cancerous cells were less sensitized by 2DG and Bifilomycin A1 to Nutlin-induced apoptosis. The indicate cell lines were treated with vehicle, Nultin, 2DG, Bifilomycin A1, and combination of Nutlin with 2DG or Bifilomycin A1 for 72 hours and analyzed for sub-G1 cells.

Supplementary Table S1: Primers for real-time PCR analysis

Gene	Forward	Reverse
ATG3	5′-ACATGGCAAT GGGCT ACAGG-3′	5′-CTGTTTGCACCGCTTATAGCA-3′
ATG5	5′-AAAGATGTGCTT CGAGATGTGT -3′	5′-CACTTTGTCAGTTACCAACGTCA-3′
ATG7	5′-ATGAT CCCTGT AACTT AGCCCA-3′	5′-CACGGAAGCAAACAACTTCAAC-3′
ATG10	5′-AGACCAT CAAAGGACT GTT CTG A-3′	5′-GGGTAGATGCTCCTAGATGTGAC-3′
ATG12	5′-CTGCTGGCGACACCAAGAAA-3′	5′-CGTGTTCGCTCTACTGCCC-3′
G6PD	5′CGAGGCCGTCACCAAGAAC-3′	5′-GTAGTGGTCGATGCGGTAGA-3′
HKII	5′-TTGACCAGGAGATTGACATGGG-3′	5′-CAACCGCATCAGGACCTCA-3′
PFK1	5′-ATTGCGGTTTT CGATGCCAC-3′	5′-GCCACAACTGTAGGGTCGT-3′
PGAM2	5′-AGAAGCACCCCT ACT ACAACT C-3′	5′-TCTGGGGAACAATCTCCTCGT-3′
β-Actin	5′-TCGT GCGT GACATTAAGGAG-3′	5′-GTCAGGCAGCTCGTAGCTCT-3′
